# Dissecting the correlates of N-terminal prohormone brain natriuretic peptide in acute infective endocarditis

**DOI:** 10.1007/s15010-022-01813-y

**Published:** 2022-04-16

**Authors:** Lorenzo Bertolino, Maria Paola Ursi, Domenico Iossa, Arta Karruli, Fabiana D’Amico, Rosa Zampino, Giovanni Dialetto, Marisa De Feo, Emanuele Durante-Mangoni, The Monaldi Hospital Cardiovascular Infection Study Group, Emanuele Durante-Mangoni, Domenico Iossa, Lorenzo Bertolino, Maria Paola Ursi, Fabiana D’Amico, Arta Karruli, Mohammad Ramadan, Roberto Andini, Rosa Zampino, Mariano Bernardo, Giuseppe Ruocco, Giovanni Dialetto, Franco Enrico Covino, Sabrina Manduca, Alessandro Della Corte, Luca S. De Santo, Antonio Carozza, Marisa De Feo, Stefano De Vivo MD, Maria Luisa De Rimini, Nicola Galdieri

**Affiliations:** 1 Department of Advanced Medical & Surgical Sciences, University of Campania ‘L. Vanvitelli’, Naples, Italy; 2 Unit of Infectious and Transplant Medicine, AORN Ospedali Dei Colli-Monaldi Hospital, Piazzale Ettore Ruggieri, 80131 Naples, Italy; 3 Unit of Cardiac Surgery, AORN Ospedali Dei Colli-Monaldi Hospital, Naples, Italy; 4 Department of Precision Medicine, University of Campania ‘L. Vanvitelli’, Naples, Italy

**Keywords:** Endocarditis, NT-proBNP, Prognostic value, Heart failure

## Abstract

**Purpose:**

To explore the prognostic value and the correlates of NT-proBNP in patients with acute infective endocarditis, a life-threatening disease, with an often unpredictable outcome given by the lack of reliable prognostic parameters.

**Methods:**

We retrospectively studied 337 patients admitted to our centre between January 1, 2006 and September 30, 2020 with available NT-proBNP level at admission. Our analyses were performed considering NT-proBNP as both a categorical variable, using the median value as the cut-off level, and numerical variable. Study end points were in-hospital mortality, cardiac surgery and 1 year survival.

**Results:**

NT-proBNP was an independent predictor of in-hospital mortality (OR 14.9 [95%C.I. 2.46–90.9]; *P* = .003). Levels below 2926 pg/mL were highly predictive of a favorable in-hospital outcome (negative predictive value 96.6%). Patients with higher NT-proBNP levels showed a significantly lower survival rate at 1 year follow-up (log-rank *P* = .005). NT-proBNP was strongly associated with chronic kidney disease (*P* < .001) and significantly higher in patients with prior chronic heart failure (*P* = .001). NT-proBNP was tightly related to staphylococcal IE (*P* = .001) as well as with higher CRP and hs-troponin I (*P* = 0.023, *P* < .001, respectively).

**Conclusion:**

Our results confirm the remarkable prognostic role of NT-proBNP in patients with IE and provide novel evidences of its multifaceted correlates in this unique clinical setting. Our data strongly support the incorporation of NT-proBNP into the current diagnostic work-up of IE.

**Supplementary Information:**

The online version contains supplementary material available at 10.1007/s15010-022-01813-y.

## Introduction

Infective Endocarditis (IE) is a life-threatening disease often complicated by acute (de novo) or acute on chronic heart failure (HF) [1], significantly impacting mortality. Recently, efforts have been done to predict the disease prognosis as accurately and quickly as possible, aiming to tailor the therapeutic approach [2, 3].

Along this path, the role of several bio-markers of systemic inflammation and/or organ damage has been investigated, including B-type Natriuretic Peptides (BNP and NT-proBNP), procalcitonin (PCT), troponin, copeptin and pro-adrenomedullin [4–6].

B-type natriuretic peptide (BNP) is synthetized and secreted by cardiomyocytes in response to mechanical strain and inflammatory stimuli [7]. Several studies have demonstrated its important role in the management of patients with HF [8–12] and sepsis [13, 14]. Thus, serum levels of this marker are currently considered key for diagnosis and risk stratification in patients with HF [15]. Moreover, its serum levels have been correlated to sepsis and sepsis-associated cardiac dysfunction [16–22].

IE is a unique disease model in this regard, as often characterized at the same time by both HF and sepsis. Indeed, these conditions represent the most common and serious complications of IE and have a major impact on prognosis [1, 12, 14].

Despite of the emerging evidences suggesting a role for BNP in predicting prognosis of patients with IE [23–26], its clinical correlates and its value for patient risk stratification in this setting remain to be established [1].

Accordingly, in this study we analyzed in depth the correlates and the prognostic value of NT-proBNP in a large sample of patients with acute IE.

## Patients and methods

### Study design

This was a retrospective study conducted at the Unit of Infectious & Transplant Medicine, Monaldi Hospital, University of Campania “Luigi Vanvitelli”, involving patients who received a diagnosis of IE admitted between January 2006 and September 2020. IE diagnosis was done according to existing criteria over time (modified Duke criteria until 2015 [27] and ESC criteria from 2016 on [1]). In the study period (2006–2020), we observed in our Hospital, a regional referral center for IE, 568 IE cases. NT-proBNP serum levels, measured on Hospital admission or a few days later in the acute phase of the disease, and before cardiac surgery, were available in 337 IE patients, who were included in this study.

This study was approved by the Ethics Committee of the University of Campania “Luigi Vanvitelli” and AORN Ospedali dei Colli. All patients gave their informed consent to the anonymous use of their clinical data.

### Patients included

Detailed data of patients were available as part of a standardized protocol of IE evaluation in use at our Unit, which includes a baseline clinical evaluation, clinical history, physical examination and demographics, chest X-ray, abdominal ultrasound scan and laboratory analyses (including C-reactive protein [CRP], creatinine, high-sensitivity cardiac troponin I [hs-troponin I] and d-dimers). According to the protocol, a trans-thoracic echocardiogram (TTE) was performed in all patients within 72 h of admission, followed by a trans-esophageal echocardiogram (TEE) where needed. Detailed information about IE characteristics (on native valve, prosthetic valve or cardiac implantable electronic device [CIED]), endocardial vegetation (size and position), and isolated causative pathogens were also collected. In terms of infection site, patients with vegetation located on more than one site were recorded as multisite. Moreover, patients with CIED endocarditis and any valve vegetation were considered as CIED-related IE. Patients with prosthetic and native valve involvement were considered as prosthetic IE.

Embolic events, defined as acute complications causing overt clinical manifestations [28], as well as valve perforation and abscesses (diagnosed by means of echocardiography and/or intra-operative findings) were also recorded. Chronic HF prior to IE onset, chronic obstructive pulmonary disease (COPD), ischemic heart disease (IHD), diabetes mellitus, liver disease, malignant neoplasia, chronic kidney disease (CKD) and renal replacement therapy (intermittent haemodialysis) were considered as the main co-morbidities. Data regarding cardiac surgery were also collected.

### Study end points

Patients were followed-up after discharge from hospital until death or loss to follow up. The study end points were in-hospital mortality and surgical treatment. Mid-term survival was assessed up to one year after discharge.

### Biochemical assays

NT-proBNP levels were measured on clinical samples obtained during the routine laboratory work up using the ElectroChemiLuminescence ImmunoAssay (ECLIA) system (Elecsys 2010 analyzer). NT-proBNP was preferred over BNP due to its more stable circulating levels and the absence of significant interaction with drugs. This assay has a lower detection limit of 5 pg/mL and a linearity range up to 35,000 pg/mL. Normal value threshold is ≤ 125 pg/mL.

### Statistical analyse

Our analyses were performed considering NT-proBNP both as a categorical variable, separating cases with NT-proBNP below or above the median value of the sample distribution, and as numerical variable. Numerical variables are presented as median and interquartile range (IQR), while categorical/nominal data are presented as number and percentage.The Mann–Whitney U test or Kruskal–Wallis test (for more than two groups) were used to assess statistical significance of the differences between numerical groups of variables whilst the Chi-square test was used to compare differences between the categorical variables. Logistic regression analyses of independent predictors of NT-proBNP levels and hospital mortality was performed by block entering in the model all variables significantly associated with each of these outcomes on the univariate analysis. Correlation between numerical values was assessed by Spearman’s coefficient. To evaluate the predictive performance of biomarker levels on IE outcome, we calculated the area under Receiver Operating Characteristic (ROC) curve, entering in-hospital mortality as the state variable.Furthermore, an analysis of 1 year survival was performed drawing Kaplan–Meier curves, assessing differences by the log-rank test.The significance level was set at 5% and all tests were 2-tailed. All analyses were performed using the statistical software for Windows Statistical Package for Social Sciences v. 22 (SPSS, Inc., Chicago, Illinois, USA).

## Results

The study cohort included 337 acute IE patients with a median age of 64 years and a high proportion of males (237 pts, 70.3%). The median NT-proBNP level was 1689.0 pg/mL. Baseline clinical characteristics of the study group are shown in Table 1. Cardiac surgery was indicated in 246 patients (73.4%) and actually performed in 208 (62.3%) subjects. Despite of an existing indication, surgery was not performed in 39 out of 337 patients (11.5%) because of high preoperative risk prediction, death before surgery or patient refusal. Embolic events occurred in 104 patients (30.9%). The in-hospital mortality rate was 9.6%.

**Table 1:** Baseline clinical features of the 337 IE cases studied

General characteristics	
Total Number	337
Age, years, median [IQR]	64 [51.5–73]
Male gender, number (%)	237 (70.3)
Female gender, number (%)	100 (29.7)
Comorbidities, number (%)	
Chronic heart failure (prior to IE onset)	101 (30)
Chronic obstructive pulmonary disease	73 (21.7)
Ischemic heart disease	66 (19.6)
Diabetes mellitus	62 (18.4)
Liver disease	61 (18.1)
Chronic kidney disease	59 (17.5)
Malignant neoplasia	49 (14.5)
Renal replacement therapy (intermittent haemodialysis)	18 (5.3)
Biochemical data, median [IQR]	
NT-proBNP, pg/mL	1689 [497–5703]
Creatinine, mg/dL	1.0 [0.8–1.3]
HS-troponin I, ng/mL	0.06 [0.02–0.65]
D-dimers, ng/mL	605 [296.5–1101.0]
C-reactive protein, mg/dL	5.3 [2.5–9.9]
Vegetation location, number (%)	
Aortic valve	115 (34.3)
Mitral valve	79 (23.6)
Tricuspid valve	18 (5.4)
Pulmonary valve	7 (2.1)
Cardiac implantable electronic device	86 (25.7)
Multivalve involvement	28 (8.4)
Other	2 (0.6)
IE subtype, number (%)	
Native valve	145 (43.3)
Prosthetic valve	93 (27.8)
Cardiac implantable electronic device	86 (25.7)
Other	11 (3.3)
IE causative pathogen, number (%)	
*S. aureus*	53 (15.7)
Coagulase negative* staphylococci*	63 (18.7)
*Streptococcus *spp*.*	97 (28.8)
*Enterococcus *spp*.*	52 (15.4)
Gram negatives	14 (4.2)
*Candida *spp*.*	2 (0.6)
Other microorganisms	7 (2.1)
Negative cultures	49 (14.5)
Cardiac surgery, number (%)	
Surgical indication (2 missing)	246 (73.4)
Surgical treatment (3 missing)	208 (62.3)
Complications, number (%)	
Embolic events	104 (30.9)
Valve perforation	29 (8.6)
Annular abscess	46 (13.6)
In hospital mortality (2 missing), number (%)	
Discharged alive	303 (90.4)
Discharged dead	32 (9.6)

**Fig. 1:**
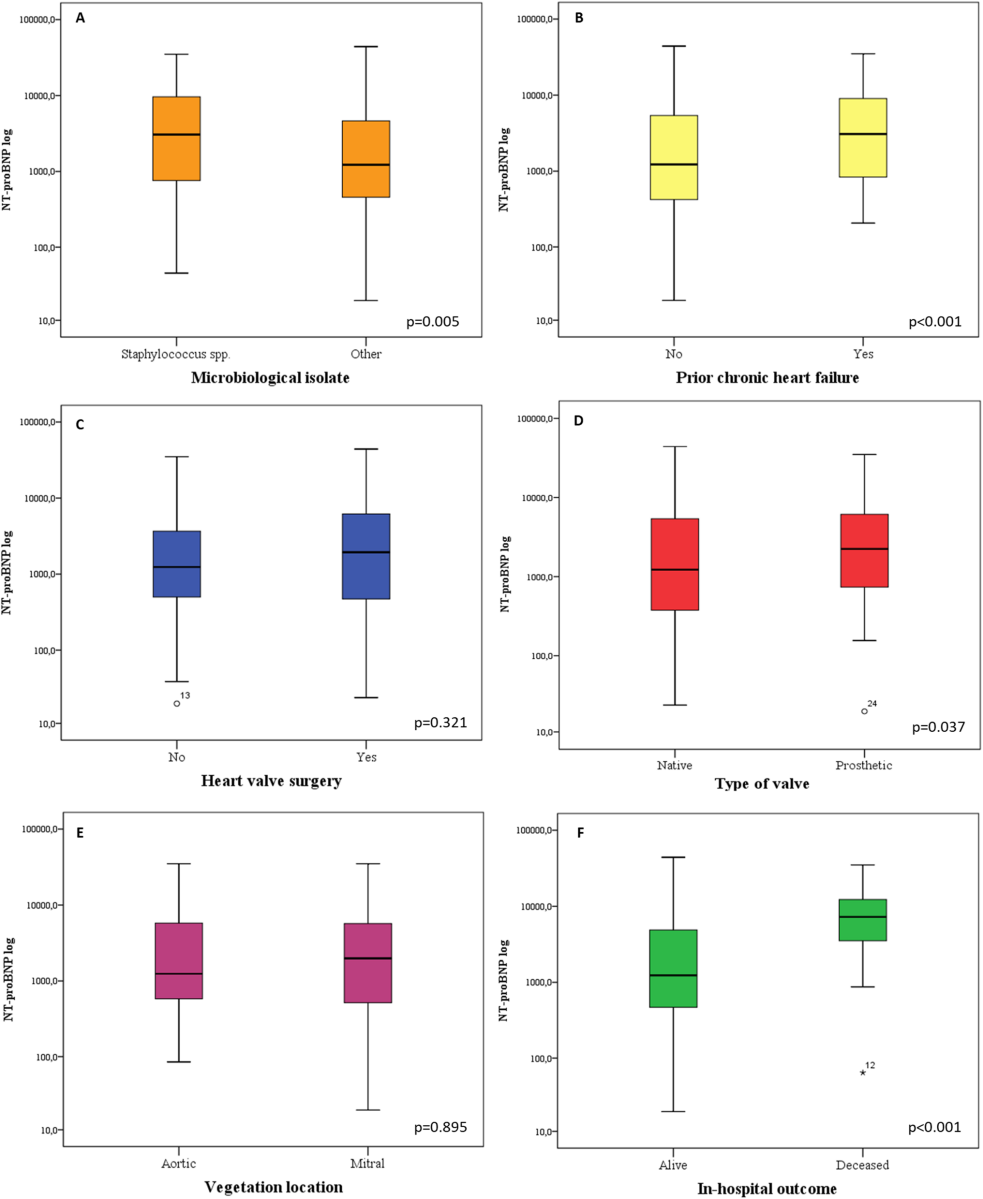
NT-proBNP levels in different IE patient subgroups (log-transformed NT-proBNP levels are shown). Each boxplot depicts the NT-proBNP median levels among each subgroup of IE patients. Panel **A** Microbiological isolates (*Staphylococcus* spp vs others). Panel **B** Prior heart failure. Panel **C** Heart valve surgery. Panel **D** type of valve (native vs prosthetic). Panel **E** Vegetation location (aortic vs mitral). Panel **F** in-hospital outcome (alive vs deceased)

On univariate analysis (Table 2), performed considering NT-proBNP as a categorical variable, we observed a statistically significant association between NT-proBNP levels and older age (*P* < 0.001). Older patients were more likely to have CHF and CKD, both conditions related to a higher NT-proBNP level (data not shown). NT-proBNP levels were also related to IE etiology (Table 2). Significantly higher levels of this biomarker were observed in staphylococcal IE (including *S. aureus* and coagulase negative staphylococci) compared with cases due to other microorganisms (*Enterococcus *spp, *Streptococcus *spp, etc.) (*P* = 0.001). The median value of NT-proBNP in staphylococcal IE was 2633.0 pg/mL, significantly higher than in IE of other etiology (1245.0 pg/mL; *P* = 0.005) (Fig. 1A). In addition, we observed a strong association of NT-proBNP with other biomarkers, such as creatinine, hs-troponin I, d-dimers and CRP (*P* < 0.001, *P* < 0.001, *P* < 0.001 and *P* = 0.023, respectively) (Table 2). Spearman correlation confirmed these findings (Supplementary Fig. 1).NT-proBNP levels increased directly with raising serum creatinine concentrations (*r* = 0.462; *P* < 0.001) (Supplementary Fig. 1A) and were also significantly higher in patients with any of the major comorbidities considered. Specifically, a strong association (*P* < 0.001) was observed with IHD and CKD (Table 2). Moreover, prior chronic HF also translated into higher NT-proBNP levels (*P* = 0.001). The median value of NT-proBNP was 2701 pg/mL in prior HF patients and 1272.5 pg/mL in those without (*P* < 0.001) (Fig. 1B).

**Table 2:** Univariate and multivariate analyses of clinical, biochemical and outcome variables associated to NT-proBNP levels in the study cohort (*n* = 337)

Parameter	Univariate analysis		*P*	Multivariate analysis	*P*
	NT-proBNP			OR [CI 95%]	
	≤ 1689.0 (*n* = 169)	> 1689.0 (*n *= 168)			
General characteristics					
Age, median [IQR]	56 [46–68.5]	69 [59–75]	< 0.001	1.046 [1.019–1.074]	0.001
Sex, *n* (%)			0.096		
M	126 (74.5)	111 (65.7)			
F	43 (25.5)	57 (34.5)			
Vegetation location, *n* (%)			0.054		
Aortic valve	64 (38)	51 (30.5)			
Mitral valve	38 (22.6)	41 (24.5)			
Tricuspid valve	14 (8.3)	4 (2.4)			
Pulmonary valve	4 (2.5)	3 (1.8)			
CIED	35 (20.8)	51 (30.5)			
Other	0 (0)	2 (1.3)			
Multisite location	13 (7.8)	15 (9)			
Heart side, *n* (%)			0.731		
Left-sided	113 (67.3)	105 (63.2)			
Right-sided	51 (30.3)	56 (33.7)			
Bilateral	4 (2.4)	5 (30.1)			
Type of valve, *n* (%)			0.145		
Native	83 (65.3)	62 (55.8)			
Prosthetic	44 (34.7)	49 (44.2)			
Microorganisms, *n* (%)			< 0.001	1.246 [0.957–1.621]	0.102
S. aureus	21 (12.4)	32 (19)			
Other *Staphylococcus*	22 (13)	41 (24.4)			
*Streptococcus* spp.	66 (39)	31 (18.4)			
*Enterococcus* spp.	28 (16.6)	24 (14.3)			
Gram negative	4 (2.4)	10 (6)			
*Candida* spp.	0 (0)	2 (1.2)			
Other microorganisms	2 (1.2)	5 (3)			
Negative culture	26 (15.4)	23 (13.7)			
Microorganisms, *n* (%)			0.001	0.249 [0.083–0.751]	0.014
*Staphylococcus* spp	43 (25.4)	73 (43.4)			
Other microorganisms	126 (74.6)	95 (56.6)			
Biochemical data, median [IQR]					
C-reactive protein, mg/dL	4.7 [2.4–8.6]	6.3 [2.9–11.7]	0.023	1.018 [0.982–1.055]	0.337
Creatinine, mg/dL	0.9 [0.7–1]	1.2 [0.9–1.7]	< 0.001	1.629 [0.986–2.690]	0.057
HS-troponin I, ng/mL	0.02 [0.01–0.19]	0.11 [0.04–2.415]	< 0.001	1.001 [0.997–1.005]	0.793
D-dimers, ng/mL	507 [251–922]	724 [356.7–1412.2]	< 0.001	1.000 [1.000–1.000]	0.893
Comorbidities, *n* (%)					
Chronic heart failure (prior to IE onset)	37 (22)	64 (38)	< 0.001	0.676 [0.311–1.469]	0.322
Ischemic heart disease	20 (11.8)	46 (27.4)	< 0.001	2.360 [0.891–6.247]	0.084
Chronic obstructive pulmonary disease	27 (15.9)	46 (27.4)	0.012	1.144 [0.487–2.686]	0.757
Diabetes	23 (13.6)	39 (23.2)	0.025	0.393 [0.145–1.061]	0.065
Liver disease	35 (20.7)	26 (15.5)	0.258		
Malignant neoplasia	18 (10.6)	31 (18.3)	0.046	2.244 [0.879–5.729]	0.091
Chronic kidney disease	4 (2.3)	55 (32.7)	< 0.001	13.339 [2.530–70.340]	0.002
Haemodialysis	0 (0)	18 (10.7)	< 0.001	N/A	N/A
Cardiac surgery^a^, *n* (%)					
Surgical indication	114 (68.2)	132 (78.5)	0.036	2.237 [0.967–5.175]	0.060
Surgical treatment	95 (56.8)	113 (67.6)	0.055		
Complications, *n* (%):					
Embolic events	54 (32)	50 (29.7)	0.724		
Valve rupture	18 (10.6)	11 (6.5)	0.244		
Annular abscess	19 (11.2)	27 (16)	0.208		
In-hospital outcome, *n* (%)			< 0.001	7.671 [1.550–37.961]	0.013
Survivors	164 (97.7)	139 (83.3)			
Non-survivors	4 (2.3)	28 (16.7)			

^a^Includes both open-heart valve surgery and CIED extraction procedures

Concerning cardiac surgery, an additional analysis was carried out excluding CIED-IE (86 pts, who underwent percutaneous lead extraction) or cases with an undefined location (4 pts). Therefore, 247 patients with valvular IE were assessed, showing a median NT-proBNP of 1500 pg/mL. The univariate analysis demonstrated no statistically significant relationship between NT-proBNP and open-heart valve surgery (*P* = 0.283 for indication; *P* = 0.371 for surgery performed). NT-proBNP median levels were 1941 pg/mL in those who underwent surgery and 1240.5 pg/mL in those who did not (*P* = 0.321) (Fig. 1C). There were no differences in NT-proBNP between cases with surgical indication but not operated, cases with indication and operated and cases without indication (data not shown).

At univariate analysis, the proportion of patients with higher NT-proBNP levels did not significantly differ between prosthetic and native valve IE (52.6% vs 42.7%; *P* = 0.145) (Table 2). However, the median value of this biomarker was 2242 [721.5–6512.5] pg/mL in the former vs 1226 [374.5–5410.5] pg/mL in the latter (*P *= 0.037) (Fig. 1D).

We then investigated whether NT-proBNP serum levels differed according with IE location (excluding cases with multivalve/multisite involvement). Patients with aortic valve IE did not show a higher level of NT-proBNP compared with mitral IE (1984 [495–5758] pg/mL vs 1245 [566–5793]; *P* = 0.895) (Fig. 1E). Similarly, there was no difference in NT-proBNP levels between right-sided and left-sided IE (Table 2).

We found no association between NT-proBNP and vegetation size (*P* = 0.974). In contrast, in valvular IE, those who had paravalvular abscesses showed significantly higher NT-proBNP levels (2954 [865–7564] vs 1245 [425–5410] pg/mL in those without abscesses; *P* = 0.036).

Patients with implantable cardioverter defibrillator IE had higher NT-proBNP levels than those with pacemaker IE (2550 [1599–6951] vs 1263 [309–3116] pg/mL; *P* = 0.003). The same was observed for hs-troponin I and creatinine (*P* = 0.032 and *P* = 0.002, respectively).

NT-proBNP was strongly associated with in-hospital mortality (*P* < 0.001) (Table 2). The median value of NT-proBNP in patients who died in hospital was significantly higher than in those discharged alive (5162 [3082–13546] vs 1488 [458–4984] pg/mL; *P* < 0.001) (Fig. 1F). In the logistic regression multivariate model, age, causative pathogen (staphylococci vs others), CKD and in-hospital mortality were independent correlates of a higher NT-proBNP level (Table 2).

In the univariate analysis considering in-hospital mortality as outcome, an association was found with type of valve (prosthetic vs native) (*P* = 0.004), surgical indication (*P* = 0.02) (but not surgical treatment [*P* = 0.450]), causative microorganism (*P* < 0.001) and levels of NT-proBNP (*P* < 0.001), d-dimers (*P* = 0.011), hs-troponin I (*P* = 0.043) and CRP (*P* = 0.022) (data not shown). In the logistic regression multivariate model, higher NT-proBNP levels were an independent predictor of in-hospital mortality (*P* = 0.003), together with type of valve involved (prosthetic vs native) and d-dimer levels (*P* = 0.005 and *P* = 0.002, respectively) (Table 3 and Supplementary Fig. 2).

**Table 3:** Multivariate analysis for predicting the occurrence of in-hospital mortality,considering NT-proBNP as a categorical variable

Parameter	Multivariate analysis	
	OR [CI 95%]	*P*
Clinical characteristics		
Type of valve	5.775 [1.722–19.363]	0.005
Native		
Prosthetic		
Biochemical data		
NT-proBNP	14.971 [2.465–90.923]	0.003
C-reactive protein	1.019 [0.967–1.075]	0.481
D-dimers	1.000 [1.000–1.001]	0.002

**Fig. 2:**
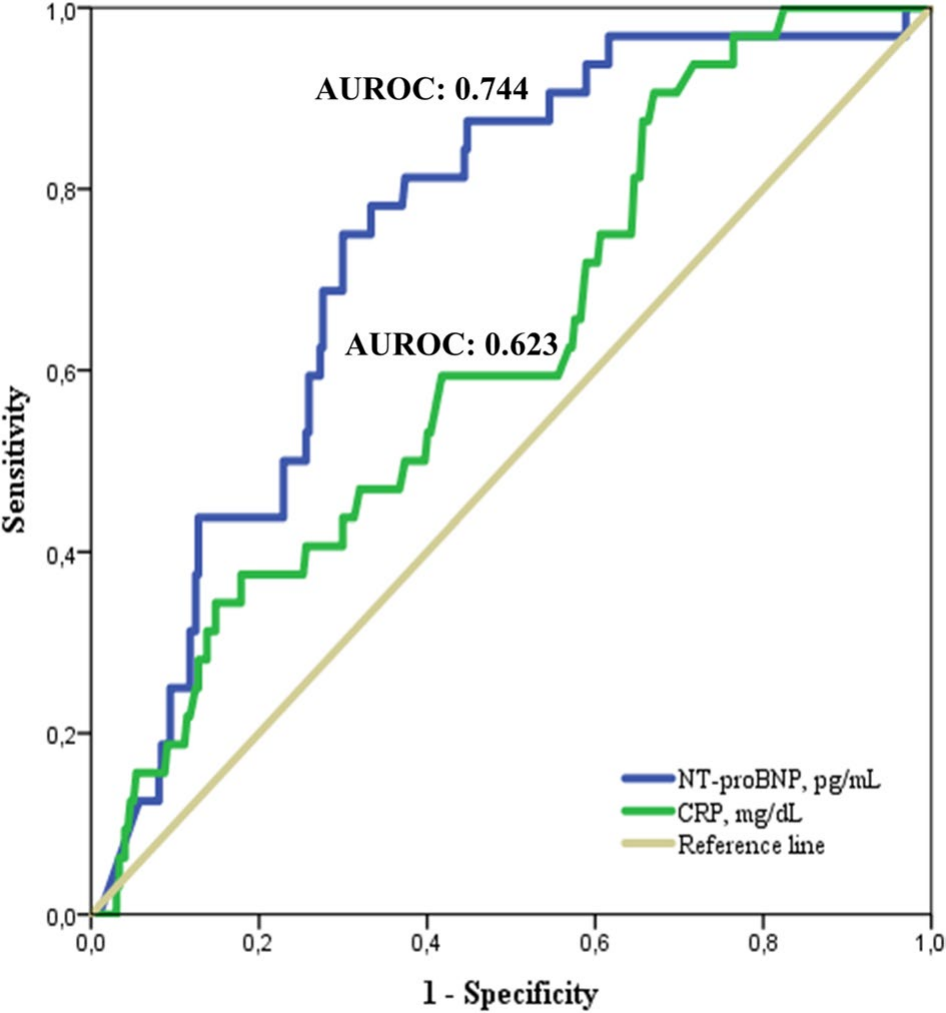
Receiver Operating Characteristic (ROC) curve analysis evaluating the predictive value of in-hospital mortality of the NT-proBNP and C-reative protein (CRP).For each of the analysed biomarker the area under the ROC curve is shown. The best cut-off for NT-proBNP (blue line) is 2926.5 pg/mL whether the cut-off for CRP (green line) is 6.1 mg/dL

The sensitivity and specificity of NT-proBNP levels in predicting in-hospital mortality were evaluated by means of a ROC curve analysis. In this analysis, an NT-proBNP cut-off value of 2926.5 pg/mL showed a sensitivity of 78.1% and a specificity of 66.7% (AUROC 0.744; [CI 95%: 0.66–0.82], P < 0.001), with a positive predictive value of 19.8% and a negative predictive value of 96.6%. Interestingly, NT-proBNP performed better when compared to CRP. Indeed, with a best cut-off of 6.1 mg/dL, CRP showed a sensitivity of 59.4% and a specificity of 58.2% (AUROC 0.623; [CI 95%: 0.52–0.71], *P* = 0.022) (Fig. 2).

**Fig. 3:**
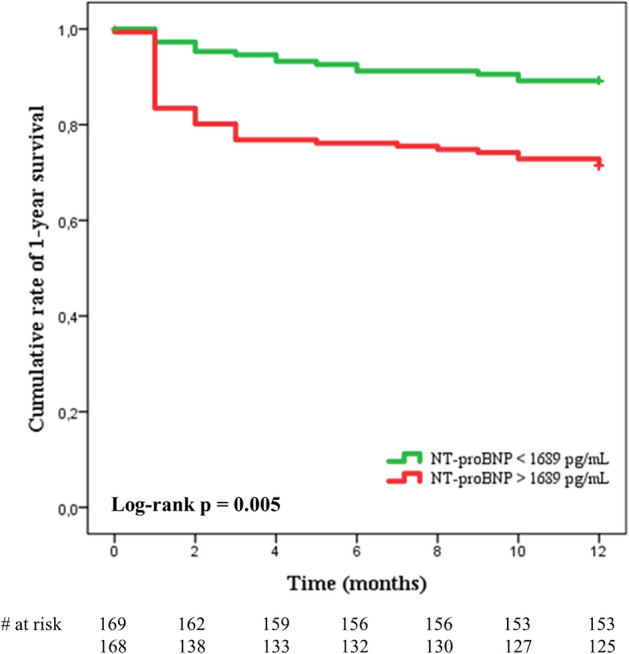
Kaplan–Meier survival curves depicting the cumulative rate of survival at 1 year according to NT-proBNP levels above or below the median total cohort value (1689 pg/mL). The number of cases at risk according to the displayed time-lime is shown at the bottom

Finally, a Kaplan–Meier survival analysis was performed to assess the influence of admission NT-proBNP levels on the 1 year survival of IE. This analysis showed that patients with NT-proBNP serum levels above the median had a significantly greater mortality rate throughout the 1-year follow-up (log rank *P* = 0.005) (Fig. 3).

## Discussion

In this study, we provide novel insights into the role of a major cardiac biomarker in IE. NT-proBNP levels showed a strong and clinically relevant prognostic role in IE, both during hospitalization and the subsequent follow-up. Levels below a defined cut off were highly predictive for a favorable outcome. Interestingly, our data suggest NT-proBNP levels correlate with markers of the infectious process rather than with drivers of IE related HF. Indeed, patients with staphylococcal infection, higher CRP and d-dimers were those showing higher NT-proBNP levels. In contrast, concentration of this biomarker did not significantly correlate with aortic valve infection—a predictor of de novo HF in IE [29]—left-sided infection or need for heart valve surgery. Neither prior HF was an independent determinant of NT-proBNP levels.

The association between NT-proBNP and age, in line with Wei et al. results [26], may be partially related to the growing rate of comorbidities, such as CHF and CKD, among the elderly. However, age was an independent predictor of increased NT-proBNP levels (Table 2), suggesting further studies are needed to explain this finding.

Our results showed that serum levels of NT-proBNP differ significantly also according with IE etiology. Indeed, higher levels were observed in patients with IE due to staphylococci, 63% of whom presented with concentrations above the median value. By contrast, most cases with streptococcal IE, well-known for a less aggressive presentation and a less ominous outcome than staphylococcal IE [30], showed a lower NT-proBNP level.

The interesting correlation between NT-proBNP and other biomarkers (hs-troponin I and d-dimers) encourages further investigation on their clinical value in patients with IE. Indeed, previous studies have already suggested a link between sepsis-induced cardiac dysfunction and troponin [16–20], as well as its role in predicting septic patient prognosis [14]. Moreover, following the profound alteration of coagulation system typically ensuing in septic patients, d-dimers may be extremely elevated [31, 32].

The significant correlation of NT-proBNP with serum creatinine levels and prior CKD should be discussed. CKD was the only comorbidity independently associated to a higher NT-proBNP. These results, consistent with current knowledge, further suggest the need for clinicians to evaluate NT-proBNP levels in light of actual kidney function [33]. However, when we performed all described analyses using an NT-proBNP value normalized for serum creatinine, findings were essentially confirmed (data not shown).

The lack of association between NT-proBNP levels and heart side involved deserves further comment. In our cohort, most patients with right-heart involvement were CIED-related IE, and were therefore carriers of a cardiac structural disorder leading to CIED placement. However, in a sub-analysis in which we excluded patients with CIED, we still did not observe significant differences between left-sided and right-sided IE in terms of NT-proBNP levels, further suggesting the association of this biomarker with inflammation rather than cardiac dysfunction.

We expected a higher level of NT-proBNP in patients requiring or undergoing cardiac surgery. In contrast, and similar to Wei et al. findings [26], patients who underwent open heart valve surgery did not show increased levels of this biomarker. Thus, at present we cannot support a role for higher NT-proBNP levels in the decision to proceed with surgery in IE patients.

An important prognostic value of NT-proBNP in IE patients emerges from this study. Indeed, the relationship between NT-proBNP levels and in-hospital mortality was statistically significant and independent of other variables. Noteworthy, 28 of 32 (87.5%) patients who died during hospitalization had NT-proBNP levels above the median value. Our results corroborate those from Wei et al. who found a similar sensitivity and specificity of NT-proBNP (76.2% and 69.1%, respectively for a cutoff value of 2260 pg/mL) [26]. Likewise, NT-proBNP prognostic value extended in the mid-term as patients with increased NT-proBNP levels on admission for IE showed a significantly lower 1 year survival.

Our results are consistent with most but not all prior literature. Kahveci et al. [23] showed a relationship between NT-proBNP and in-hospital mortality, HF, creatinine and troponin on a smaller cohort. By contrast, these investigators did not observe any association between NT-proBNP and CRP or Staph. aureus IE. Interestingly, they describe an association between this biomarker and end-systolic left ventricular diameter, end-diastolic left ventricular diameter and other echocardiographic parameters that, unfortunately, were not available in our patient cohort.

Siciliano et al. [25] found that BNP correlated with HF, a larger vegetation (≥ 10 mm), Staph aureus IE, CRP, diabetes mellitus and creatinine. In contrast, Shiue et al. [24] did not show any relationship between BNP and outcome, HF, creatinine and diabetes, but only with right-sided IE, severe aortic and mitral regurgitation, paravalvular abscesses and troponin. Some of these differences might be related to the different biochemical properties of BNP and NT-proBNP. Indeed, the latter has a longer half-life (25–120 min) and is biologically inactive [34], possibly reflecting HF and/or sepsis more accurately than BNP.

Limitations of this study include its retrospective design, the absence of recorded echocardiography data and the relatively low in-hospital mortality of the study sample.

In conclusion, our results confirm the remarkable prognostic role of NT-proBNP in patients with IE and provide novel evidences of its multifaceted correlates in this unique clinical setting. Our data strongly support the incorporation of NT-proBNP into the current diagnostic work-up of IE.

## Supplementary Information

Below is the link to the electronic supplementary material.

Supplementary file1 (PDF 257 KB)
