# Supplementary material for: Dissecting the correlates of N-terminal prohormone brain natriuretic peptide in acute infective endocarditis
Source: Infection. 2022 Apr 16;50(6):1465–74. doi: 10.1007/s15010-022-01813-y (PMC9705495; doi:10.1007/s15010-022-01813-y)
Supplement: Supplementary file 1 — Supplementary file1 (PDF 257 KB) [file 15010_2022_1813_MOESM1_ESM.pdf]

Dissecting the correlates of N-terminal prohormone brain natriuretic peptide in acute infective endocarditis - Infection

Lorenzo Bertolino[a], Maria Paola Ursi[a], Domenico Iossa[a], Arta Karruli[a], Fabiana D'Amico[a], Rosa Zampino[a,b], Giovanni Dialetto[c], Marisa De Feo[c], Emanuele Durante-Mangoni[b,d]; on behalf of the Monaldi Hospital Cardiovascular Infection Study Group (see Appendix)

[a]Department of Advanced Medical & Surgical Sciences, University of Campania 'L. Vanvitelli', Napoli, Italy
[b]Unit of Infectious and Transplant Medicine, AORN Ospedali dei Colli-Monaldi Hospital, Napoli, Italy
[c]Unit of Cardiac Surgery, AORN Ospedali dei Colli-Monaldi Hospital, Napoli, Italy
[d]Department of Precision Medicine, University of Campania 'L. Vanvitelli' Napoli, Italy

Corresponding author:
Emanuele Durante Mangoni, MD PhD
Ospedale Monaldi, Piazzale Ettore Ruggieri
80131 Napoli, Italy
Ph: +39.081.7062475
Fax: +39.081.7702645
Email: emanuele.durante@unicampania.it

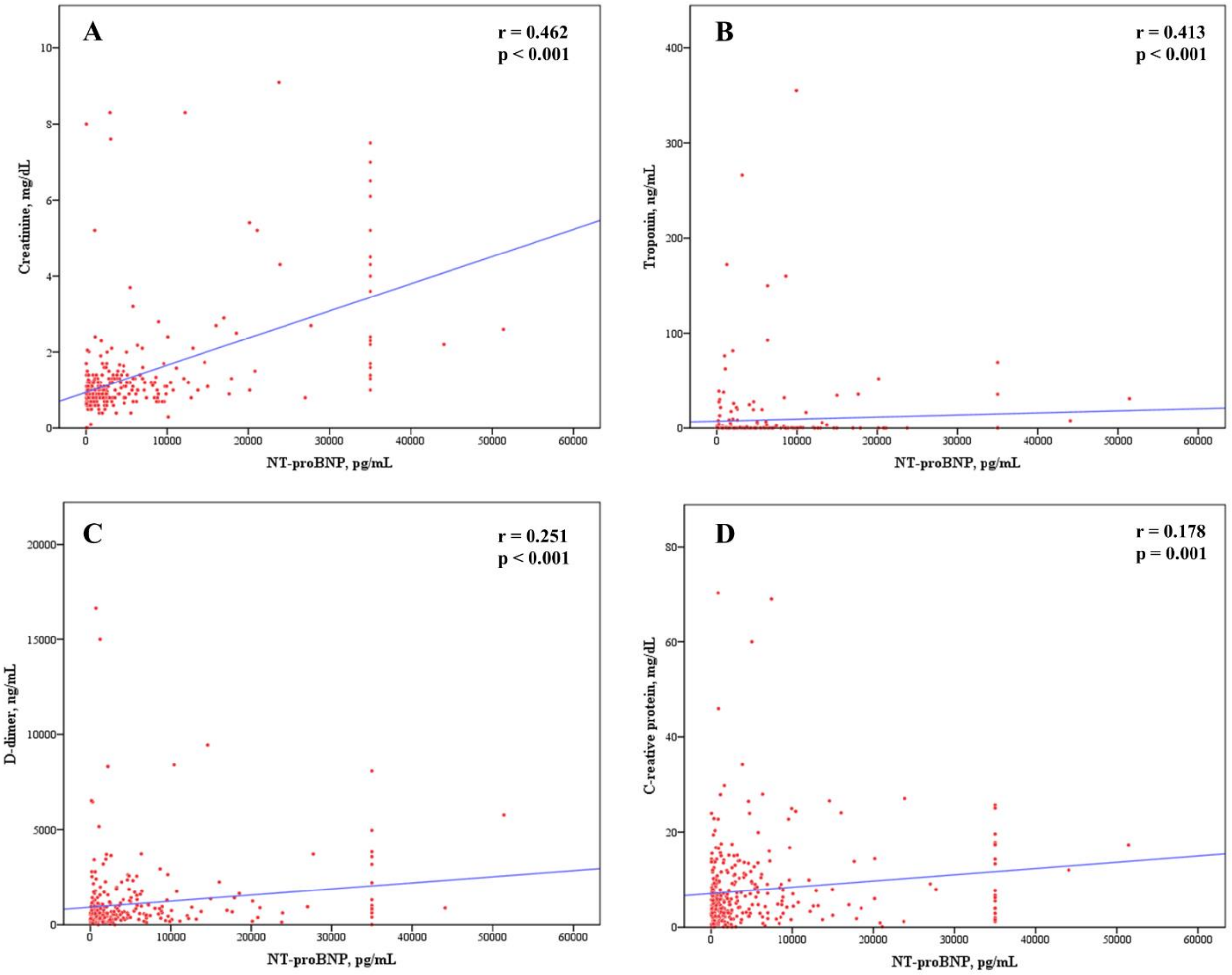

Supplementary Fig 1 Spearman correlation analyses of NT-proBNP levels with other biomarkers. (A) Correlation between NT-proBNP and creatinine, (B) Correlation between NT-proBNP and hs-troponin I, (C) Correlation between NT-proBNP and d-dimers, (D) Correlation between NT-proBNP and C-reactive protein

Color in print for this figure is not necessary

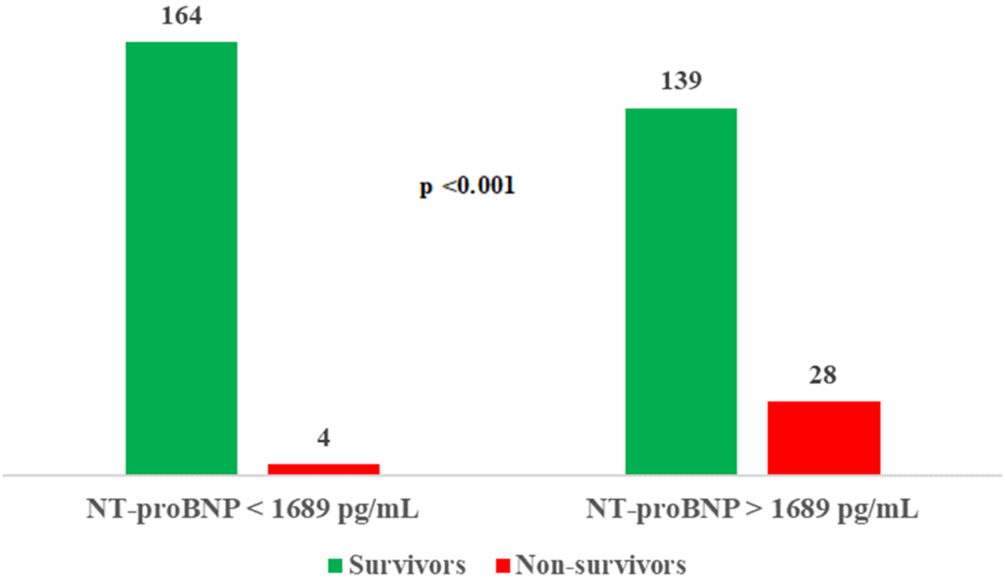

Supplementary Fig 2 Bar chart showing the number of cases with positive or negative in-hospital outcome according to NT-proBNP median value

Color in print for this figure is not necessary
